# Actinic keratoses: review of clinical, dermoscopic, and therapeutic aspects^☆^^☆☆^

**DOI:** 10.1016/j.abd.2019.10.004

**Published:** 2019-11-06

**Authors:** Clarissa Prieto Herman Reinehr, Renato Marchiori Bakos

**Affiliations:** Department of Dermatology, Hospital de Clínicas de Porto Alegre, Porto Alegre, RS, Brazil

**Keywords:** Dermoscopy, Keratosis, actinic, Neoplasms, squamous cell, Precancerous conditions, Skin neoplasms

## Abstract

Actinic keratoses are dysplastic proliferations of keratinocytes with potential for malignant transformation. Clinically, actinic keratoses present as macules, papules, or hyperkeratotic plaques with an erythematous background that occur on photoexposed areas. At initial stages, they may be better identified by palpation rather than by visual inspection. They may also be pigmented and show variable degrees of infiltration; when multiple they then constitute the so-called field cancerization. Their prevalence ranges from 11% to 60% in Caucasian individuals above 40 years. Ultraviolet radiation is the main factor involved in pathogenesis, but individual factors also play a role in the predisposing to lesions appearance. Diagnosis of lesions is based on clinical and dermoscopic examination, but in some situations histopathological analysis may be necessary. The risk of transformation into squamous cell carcinoma is the major concern regarding actinic keratoses. Therapeutic modalities for actinic keratoses include topical medications, and ablative and surgical methods; the best treatment option should always be individualized according to the patient.

## History and definition

Actinic keratoses, also called solar or senile keratoses, were described by Dubreuilh in 1826.^1^, ^2^ Later, the term “keratoma senilis” was proposed by Freudenthal, and in 1958 Pinkus renamed the lesions as actinic keratoses.^3^ Although classically categorized as pre-neoplastic lesions, some authors suggest considering them as *in situ* neoplasms, since they derive from clonal DNA modifications in keratinocytes.^2^, ^4^, ^5^, ^6^, ^7^ In this sense, actinic keratoses are considered as having characteristics of malignancy since their genesis, both from the standpoint of cytological alterations presented by epidermal keratinocytes, which are similar to those observed in spinocellular carcinomas (SCCs), including loss of polarity, nuclear pleomorphism, dysregulated maturation, and increased number of mitoses, as well as from the molecular standpoint, presenting identical mutations in the p53 protein.^3^ The difficulty in establishing unambiguous criteria for determining when an actinic keratosis undergoes SCC transformation reinforces this hypothesis. According to Ackerman, there is no clear threshold between actinic keratoses and thin SCCs, and actinic keratosis are considered a part of the evolutionary spectrum of SCC, described as an “embryonic” SCC.^2^ Therefore, proposed nomenclatures replacing the term actinic keratosis would include keratinocytic intraepidermal neoplasia and intraepidermal solar keratotic SCC.^3^

Actinic keratoses are formed by proliferation of keratinocytes with varying degrees of dysplasia in the epidermis, *i.e*., they represent intraepithelial keratinocytic dysplasias; besides, they have a potential for malignant transformation into non-melanoma skin cancer (NMSC), especially in the case of SCC, and they occur preferentially in sun exposed areas.^1^, ^8^, ^9^

## Epidemiology

Actinic keratoses represent the third reason for dermatological consultation, losing only to acne and dermatitis.^10^ With the overall aging of the population, a gradual increase in the frequency of actinic keratoses is expected.^10^ Regarding the prevalence of actinic keratoses, the World Health Organization estimates that the highest levels are observed in Caucasians living close to the Equator.^11^

In the international scenario, the prevalence of actinic keratoses is higher in Australia, where fair skin type individuals are predominant and high exposure to UV radiation occurs, followed by the United States and Europe.^12^ The prevalence of actinic keratoses ranges from 40% to 60% in Australia among Caucasians over 40 years of age, and 11.5% to 26% in the United States in individuals over 30 years of age.^13^, ^14^, ^15^, ^16^, ^17^, ^18^ In England, a population-based study observed a prevalence of actinic keratoses of 15.4% in men and 5.9% in women over 40 years; this prevalence was elevated to 34.1% and 18.2% for men and women, respectively, when only patients older than 70 years were considered.^19^ In a Spanish study, the prevalence of actinic keratoses was 28.6% in patients above 45 years; this prevalence was higher in men than in women and the values increased according to age for both sexes.^20^ Another study, carried out in Austria, found a prevalence of actinic keratoses of 31% in patients over 30 years of age; the prevalence was higher in men than in women, and increased according to age for both sexes (39.2% in males *vs.* 42.3% in females).^21^ Finally, in the Asian population, studies have demonstrated a lower prevalence of actinic keratoses: in South Korea, values vary from 0.02% in patients aged 40 years, 0.09% in patients aged 60 years, and 0.21% in patients aged 70 years^22^; in China, a population-based study (1,590,817 patients evaluated) observed a prevalence of 0.52%, with a mean age of 69.8 ± 11.8 years.^23^

In Brazil, actinic keratoses represent the fourth most common dermatological diagnosis.^1^ In addition, they represent the main reason for dermatological consultation in Brazil in individuals over 65 years (17.2%); in Southern Brazil, this corresponds to 7.4% of the diagnoses and in the North region, to 2.89% of visits.^24^ In a study conducted in Curitiba with 491 patients, with a mean patient age of 59.8 years, the prevalence of actinic keratosis was 60.79% in women and 30.9% in men.^25^ Another study, conducted in Bauru, evaluated the prevalence of actinic keratoses only in Japanese descendants living in Brazil; the study observed a prevalence of 13.4%, with a mean age of 68.9 years; this prevalence is higher than that observed in individuals of the same ethnic composition living in Japan.^26^

As mentioned above, the prevalence of actinic keratoses increases according to the age of the patients, ranging from <10% in Caucasians aged 20–29 years, to 80% in individuals aged 60–69 years.^27^ Exceptions occur in albinos and patients carrying other genodermatoses that present defects in DNA repair genes, such as xeroderma pigmentosum, Rothmund–Thompson syndrome, Cockayne's syndrome, and Bloom's syndrome, which may present lesions in the first decade of life, and lesions with greater aggressiveness and risk.^1^, ^28^, ^29^, ^30^ Age is an independent risk factor for the development of actinic keratoses, with odds ratios (OR) ranging from 1.6 to 41.5 according to age; the OR is of 4.8 for individuals between 46 and 60 years and up to 41.5 years in individuals over 70.^31^, ^32^, ^33^, ^34^

Men have a higher prevalence of actinic keratoses, with an OR of 1.7–3.9, due to the higher average UV exposure to which men receive during life.^31^, ^32^, ^34^, ^35^, ^36^

Populations whose ethnic composition predominantly present individuals with fair skin (types I and II), who are more susceptible to the carcinogenic effects of UV radiation, also present a higher risk of developing actinic keratoses, with an OR of 1.7–6.9.^31^, ^32^, ^34^, ^35^, ^36^ In addition, geographical location is also of great importance because it represents the rate of UV radiation that a given population is exposed to and may even modify the prevalence rates in populations that have migrated, as observed is the study carried out with Japanese descendants in Bauru.^26^

Few studies evaluating the incidence of actinic keratoses are available. The first was held in Maryborough/Australia (37° S), in 1986, with 1040 individuals over 40 years. In the study all patients were evaluated twice in a 12-month period. In the baseline evaluation, 59% of the subjects had actinic keratosis; in the follow-up, 60% presented new lesions. Among the patients without lesions in the baseline evaluation, 19% developed lesions observed at follow-up.^37^ A population study conducted in Wales with 1034 individuals over 60 years of age observed an incidence rate of actinic keratoses of 149 lesions per person-year and a prevalence of 23%.^38^ Another study, conducted in South Korea, evaluated 77,975 individuals with actinic keratoses above 40 years who had consulted with dermatologists between 2006 and 2015 at least twice over a one-year period.^22^ The incidence rate in the ranged from 17.95 per 100,000 person-years in 2006 to 53.99 per 100,000 person-years in 2015, these values were lower than the expected rates in the Western population. In addition, the authors observed an increase in the incidence rate as the patient's age increased, and this increase was higher in the 70-year age range.^22^

## Pathogenesis

The etiology of actinic keratoses involves both individual and environmental factors.^13^

Excessive exposure to UV radiation is the major factor, acting as a complete carcinogen, both inducing and promoting tumor expansion.^8^, ^39^, ^40^ UV radiation activates molecular signaling cascades that result in modifications of regulatory cytokines levels, immunosuppressive effects, and defective cell differentiation and apoptosis.^8^ UV radiation is divided into UVA, UVB, and UVC; about 94–97% of the radiation that reaches Earth's surface is composed of UVA rays, UVB rays are partially absorbed by the ozone layer and represent only 3–6%, and UVC rays are filtered by the ozone layer in the atmosphere and only minimum levels reach the Earth's surface.^8^

UVA radiation (320–400 nm) penetrates the skin more deeply and stimulates reactive oxygen species production, which damage cell membranes, their nuclei, and proteins^41^; in addition, UVA promotes guanine (G) to thymine (T) replacement mutations in DNA.^42^ As a result, signal transduction and cellular interaction pathways are affected and abnormal cell proliferation occurs.^8^

UVB radiation (290–320 nm) is absorbed by cellular DNA, promoting errors in the repair of cyclobutane pyrimidine dimers and production of 6–4 photoproducts, as well as characteristic cytosine–thymine (C–T) DNA substitutions.^41^ These effects result in mutations in the p53 protein, which regulates the cell cycle and acts on DNA damage repair, mutations in the telomerase gene, and increase of proinflammatory cytokine production.^42^, ^43^

**Figure 1:**
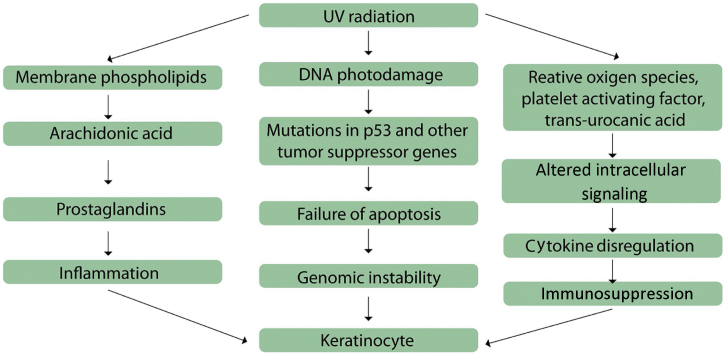
Mechanisms involved in actinic keratoses pathogenesis. Adapted from: Berman B, Cockerell CJ. Pathobiology of actinic keratosis: ultraviolet-dependent keratinocyte proliferation. Berman B, et al. (2013).^8^

Thus, mechanisms involved in the onset of actinic keratoses include inflammation, oxidative stress, immunosuppression, impaired apoptosis, cell cycle deregulation and cell proliferation, and tissue remodeling (Fig. 1).^8^

The inflammatory process is mediated by the arachidonic acid pathway, by the production of proinflammatory cytokines, and by the activation of mast cells and inhibitory factor of macrophage migration; the results of the activation of these mediators include lipid peroxidation, increase in intralesional levels of T lymphocytes and Langerhans cells, increase of p53 and Bcl-2, and reduction in Fas (cd95) and Fas-ligand, which are important initial factors in the apoptosis process of UV-mutated cells.^8^ A link between inflammation and actinic keratoses development is observed in lesions that have progressed to SCC; in some cases actinic keratoses undergo an inflammatory phase before becoming invasive.^44^ This is corroborated by the fact that anti-inflammatory therapies are effective in the treatment of actinic keratoses.^45^

Oxidative stress is also involved in the photocarcinogenesis process, as a result of the excessive exposure to ultraviolet radiation, which leads to the production of reactive oxygen species and culminates with lipid peroxidation and cell destruction, with damage to genomic and mitochondrial DNA.^8^ Altered signal transduction pathways result from membrane tyrosine kinase phosphorylation, alterations in the epidermal growth factor, in the Ras and RAF, and in the dissociation of the nuclear factor κB from the inhibitory B complex.^46^, ^47^, ^48^, ^49^ These events result in the production of cytokines, including interleukin (IL)-1, tumor necrosis factor, and IL-6, and in the activation of the arachidonic acid pathway. The final result is the shift of transcription factors to the cell nuclei, with gene expression modifications.^50^

Apoptosis disorders occur by suppression, elimination, or activation of apoptotic mediators, such as CD95 and tumor necrosis factor-associated apoptosis, and of pro-apoptotic tumor suppressor genes, as well as by regulation of p53 apoptotic activity.^51^, ^52^ Moreover, mutation of the p53 tumor suppressor gene induced by UVB radiation occurs in an early stage in cutaneous tumorigenesis.^8^

The five most important independent risk factors for actinic keratoses development are age, sex, phototypes I and II, previous history of cutaneous neoplasms, and sun exposure due to occupational reasons.^34^ The history of previous skin neoplasms (OR = 6.47) is important because it reflects the association of individual genetic factors, which may influence the sensitivity to UV radiation, and the degree of chronic UV radiation exposure to which the individual has been exposed during life.^33^, ^34^ When assessing the impact of occupational sun exposure for actinic keratoses development, workers from outside areas present a risk two-to-three times higher of developing actinic keratoses and are at increased risk for all cutaneous neoplasms, with an OR of 3.45 for actinic keratoses, 3.67 for SCC, 3.32 for basal cell carcinoma (BCC), and 1.97 for melanoma (*p* < 0.005).^53^, ^54^ Other risk factors for actinic keratoses include episodes of painful sunburn before the age of 20 years (OR = 1.21), not using sunscreen (OR = 1.81), and a positive family history for cutaneous neoplasms (OR = 1.85).^34^ Episodes of painful sunburn before 20 years of age could represent the initiating events of the carcinogenesis process, since both acute and chronic UV radiation exposure can lead to mutations in the p53 gene and subsequent clonal keratinocytic expansion.^55^

Patients with chronic use of systemic immunosuppressive drugs are a specific risk group for developing cutaneous neoplasias and dysplasias as a result of UV radiation carcinogenic effects.^56^ In solid organ transplant patients, NMSC is the most prevalent neoplasm, occurring in 27% of them.^56^, ^57^ Besides, immunosuppressed patients have a higher prevalence of actinic keratoses and a higher risk of progression of these lesions to SCC.^58^ In as study conducted in Queensland, Australia, with 495 renal and hepatic transplant patients, mean age of 54 years, and mean time of immunosuppression of 8.9 years, the authors observed the presence of actinic keratoses in 80% of the sample, and 30% of the patients had more than five lesions.^59^ The prevalence of NMSC in immunosuppressed patients is higher than in the general population and these patients have a higher risk of progression of their actinic keratoses to SCC (the incidence of SCC in immunosuppressed patients is 65 times higher than in the general population), and their SCCs are at higher risk of progression to stage IV (occurrence of metastases in 0.5–5% in the general population *vs.* 8% in immunosuppressed patients).^58^, ^59^, ^60^, ^61^ Time of immunosuppression is the most important factor for the increased risk of developing NMSC in these patients, and lesions tend to occur on field cancerization areas, with an OR of 93 for SCC development *vs.* 20-fold in patients with isolated actinic keratoses.^61^, ^62^

The development of actinic keratoses in immunosuppressed patients involves the factors previously described and aspects related to immunosuppressive medications that may even act as carcinogens, *e.g.*, azathioprine. The medication causes direct damage to the DNA when the patient is exposed to UVA radiation, in addition to being photosensitizing. In the case of cyclosporin, carcinogenic effects occur through up-regulation of TGF-β. The scientific evidence available demonstrates an increased risk of developing SCC in patients using azathioprine, cyclosporine, tacrolimus, prednisolone, and mammalian target of rapamycin (m-TOR) inhibitors, such as sirolimus and everolimus. However, patients taking m-TOR inhibitors have a 51% lower risk of developing SCC when compared to patients taking cyclosporine or tacrolimus.^63^ In addition, chronic immunosuppressive status affects the correction pathways of pre-oncogenic mutations.^57^ The role of human papillomavirus in the skin carcinogenesis of immunosuppressed patients remains controversial and the proposed mechanism is not clear.^63^ The risk of developing NMSC in the first five years after transplantation has significantly reduced in patients undergoing solid organ transplantation from 1983–1987 to 2003–2007. In the Norwegian population, the incidence of SCC was 102-fold higher than that observed in the general population in the former period, reducing to 21.6 times in 2003–2007.^64^ The implementation of individualized and less aggressive immunosuppressive protocols, periodical clinical follow-up of these patients, as well as education about sun safety habits were responsible for this decrease.^57^, ^64^

Additional features considered to be risk factors for actinic keratoses development include facial telangiectasias, ephelides, solar lentigos (OR = 1.6),^65^ solar elastosis (OR = 4.4), cutis rhomboidalis nuchae (OR = 2.9), and ≥10 melanosis on the dorsa of the hands (OR = 6).^31^, ^65^

## Clinical and histological features

**Figure 2:**
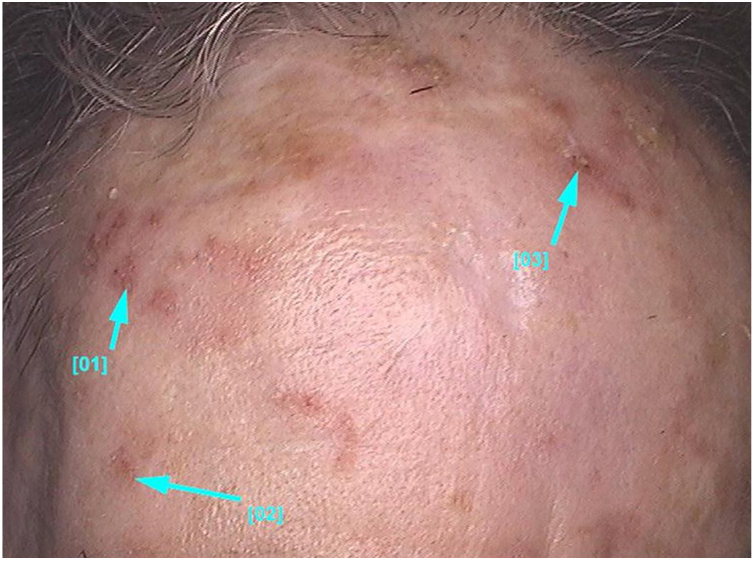
Non-pigmented facial actinic keratoses: multiple small papules and erythematous plaques with whitish scales on the surface and varying degrees of hyperkeratosis.

Actinic keratoses present as erythematous macules, papules, or plaques, usually with poorly defined borders, and they may be covered by adherent dry scales. Sometimes they are better identified by palpation than by visual inspection, and they can present varying degrees of hyperkeratosis.^1^, ^66^ The lesions are either single or multiple (Fig. 2) and their color may vary from pink to erythematous or brownish, in the case of pigmented actinic keratoses.^67^, ^68^ Infiltration degree can also be variable according to the intensity and to the extent of lesion dysplasia. They are asymptomatic in most of the cases, although some patients refer to the experience of discomfort, such as burning, pain, bleeding, and pruritus.^1^, ^66^, ^69^

Actinic keratoses predominantly occur in chronic photoexposed skin areas, such as the face, scalp in the bald area, neck, cervical region, shoulders, forearms, and back of the hands.^13^ In both sexes, the lesions tend to occur most commonly in the upper limbs, and the face and scalp regions.^13^ These regions, especially the head, neck, and forearms, are responsible for 75% of the reported lesions.^15^

Actinic keratoses can manifest in different forms and present clinical variants, such as hyperkeratotic actinic keratosis, atrophic, pigmented lichenoid actinic keratosis, cutaneous horn, and actinic cheilitis. Different variants present specific clinical and morphological characteristics and, consequently, their recognition is necessary for correct management, since certain subtypes of actinic keratoses respond better to some therapeutic modalities, as shown below (Table 1).^68^

**Table 1:** Clinical variants of actinic keratoses and their usual manifestations.

Cutaneous horn	Conical projection, markedly hyperkeratotic, white to yellowish in color, erythematous papular base with varying degrees of infiltration^70^
	Differential diagnosis with squamous cell carcinoma^68^
Actinic cheilitis	Reddish and/or leukoplastic plaque with desquamation, fissures, ulcerations, and/or areas of focal hyperkeratosis on the lips (95% of the cases in the lower lips)
	Differential diagnosis with squamous cell carcinoma^71^
Pigmented actinic keratosis	Scaly papules or plaques, rough with brown and black color^66^
	Differential diagnosis with lentigo maligna
Lichenoid actinic keratosis	Pink plaque or papule
	Usually on the upper extremities or trunk
	Differential diagnosis mainly with basal cell carcinoma^66^

Actinic keratoses may be multiple and poorly delimited in patients with severe photodamage; sometimes in such cases the lesions cannot be counted. In these situations so-called field cancerization is observed, characterized by pre-neoplastic alterations of the epithelium after long exposure to carcinogenic agents, in particular UV radiation; the field cancerization consists of lesions in different phases, from subclinical actinic keratoses to SCC.^72^, ^73^ Field cancerization was described by Slaughter in 1957, by analysis of the perilesional stratified squamous epithelium of SCC in the oral mucosa; Slaughter reported histological changes in specimens of skin adjacent to SCC, such as cellular atypia and even SCC *in situ*, although clinically the skin was unchanged.^73^ This concept of multiple contiguous foci of altered clonal keratinocytes in the field cancerization represents the set of alterations found in chronic photodamaged skin with multiple NMSC, including multiple actinic keratoses; in this case the perilesional skin without apparent clinical alterations may present cytogenetic modifications associated with carcinogenesis.^1^, ^74^ The concept of field cancerization corroborates the chronic course of actinic keratoses, with frequent recurrences. It also reinforces the need to treat the whole field cancerization area to achieve long-term remission. Contrastingly, if only visible lesions are treated, adjacent mutated areas may further develop new lesions.^74^

Although the diagnosis of actinic keratoses is based on clinical examination, in some cases a skin biopsy is necessary; these are the major criteria for biopsies: large lesions (>1 cm in diameter), bleeding, ulceration or induration, rapid lesional growth and erythema. The minor criteria are intense lesional pruritus, pain, pigmentation, hyperkeratosis, and palpable lesion; in addition, absence of response to usual treatments and presence of some unusual characteristics may also be associated with the progression of actinic keratoses to SCC and indicate the need for histopathological examination.^3^, ^5^, ^68^, ^75^, ^76^

It should be noted that SCCs that in mucosal areas present a higher risk of progression; for this reason, early clarification of the etiology of labial lesions is essential. In this context, performing a biopsy and sending the material for histopathological analysis is mandatory. Performing a cutaneous biopsy is imperative in cases of suspected SCC: when there is ulceration, increased semimucosa thickness, lip texture changes, or loss of definition between the transition from labial commissure to adjacent skin.^71^

## Classifications

Different proposals for the classification of actinic keratoses have been described. They take into account clinical, dermatoscopic, and histopathological aspects, whether isolated or associated (Table 2). Initially, Olsen et al. classified the lesions in three grades according to clinical aspects. They recommended assessing the severity of the lesions in clinical practice.^76^, ^77^ Another classification –proposed by the 2015 European Guideline for the treatment of actinic keratoses and created by Röwert-Huber et al. – orders the lesions according to histological examination from grade I to grade III.^6^, ^76^ It is important to emphasize that the degree of agreement between the clinical and histological graduation is low, which reinforces that every actinic keratoses should be treated, independently of their grade.^78^ In addition to the grading systems mentioned above, others are available, including Cockerell's, which associates clinical examination and degree of keratinocyte atypia and Goldberg's, which classifies them as proliferative or non-proliferative based on clinical behavior and histological characteristics.^3^, ^79^, ^80^ Moreover, Zalaudek et al. used dermoscopy to classify facial actinic keratosis. Based on histopathological examination, it is also possible to categorize actinic keratoses into seven subtypes: hypertrophic, atrophic, bowenoid, acantholytic, epidermolytic, lichenoid, and pigmented.^1^, ^7^, ^81^ In this grading system an overlap of histological subtypes may occur in a single lesion.^43^ Finally, there is a lack of consensus in the validation of these classifications. Their use remains controversial and it is still not possible to define a gold-standard grading system for actinic keratoses in clinical practice.^82^

**Table 2:** Existing classifications for actinic keratoses.

Publications in chronological order	What the classification is based on
Olsen et al. (1991)^77^	Clinical examination
	Grade I: easily palpable and barely visible
	Grade II: easily visible and palpable
	Grade III: visible and hyperkeratotic
Goldberg et al. (1994)^80^	Clinical examination and evolutionary characteristics
	Proliferative lesions: resistant to therapy, tendency to growth and progression to SCC
	Non-proliferative
Cockerell (2000)^3^	Clinical and histological examination
	Grade I: flat macula without hyperkeratosis, including subclinical, histopathology with atypia of keratinocytes in the lower third of the epidermis
	Grade II: hyperkeratotic lesion with variable induration, histopathology with atypia in the lower two-thirds of the epidermis
	Grade III: indurated plaques, may be pigmented, intense atypia throughout the epithelium
Röwert-Huber et al. (2007)^6^	Histological findings
	Grade I: atypical keratinocytes in the basal and suprabasal layers of the epidermis
	Grade II: atypical keratinocytes in the lower two-thirds of the epidermis
	Grade III: atypia superior to two-thirds of the epidermis and involvement of the adnexal epithelium
Zalaudek et al. (2014)^79^	Dermatoscopic findings
	Grade I: erythematous pseudonetwork, discrete scales
	Grade II: erythematous pseudonetwork, keratotic and enlarged follicular openings
	Grade III: hyperkeratosis with thick scales or enlarged keratotic follicular openings associated with scales

SCC, squamous cell carcinoma.

## Diagnosis

Actinic keratoses are diagnosed clinically in the majority of the cases. Lesions presenting compatible history data and physical examinations may be recognized and do not need complementary analyses. Dermoscopy has been shown to be extremely important in increasing the level of confidence and accuracy in equivocal lesions. Other noninvasive imaging methods, such as confocal microscopy (CM), may also be useful in specific situations when available. Finally, doubtful cases will require histopathological study to confirm the diagnosis.

To know the clinical characteristics of the main differential diagnoses of actinic keratoses and how to use the auxiliary methods for diagnosis is crucial in this process.

### Dermoscopy

Dermoscopy is a fast-performing, noninvasive method that helps in the diagnosis of actinic keratoses and allows them to be differentiated from their differential diagnoses; moreover, actinic keratoses have well established dermatoscopic criteria. Dermoscopy has high sensitivity and specificity for the diagnosis of actinic keratoses, with values of 98.7% and 95%, respectively.^83^, ^84^

**Figure 3:**
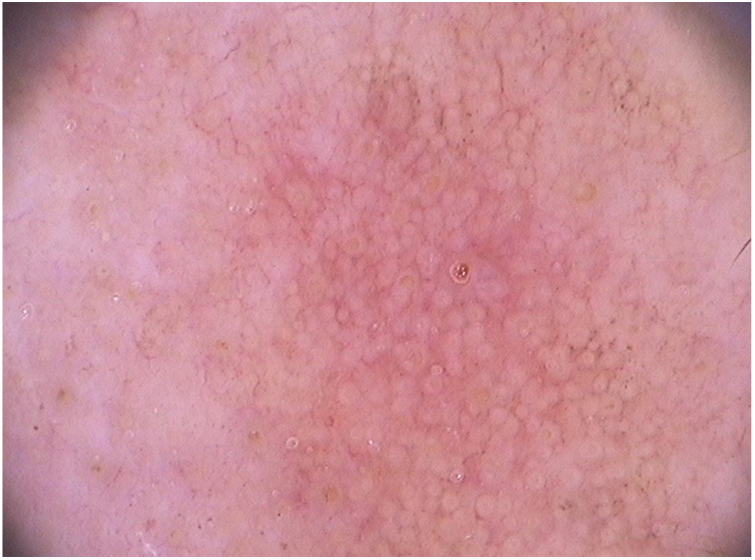
Dermoscopic image (FotoFinder®, x20) of the “strawberry” pattern observed in facial actinic keratosis.

In facial actinic keratoses four dermoscopic findings are described as essential: (1) erythema forming a pink-reddish vascular pseudonetwork surrounding hair follicles, (2) yellowish-white scales, (3) thin and wavy vessels surrounding the follicles, and (4) follicular openings filled with keratotic plugs.^83^ These structures define the so-called “strawberry” pattern described for most of the facial actinic keratoses (Fig. 3).^83^ Anther finding is the “rosette” sign, seen only with polarized light dermoscopy, a figure resembling a four-leaf clover, formed by four whitish points surrounding the follicular opening.^85^, ^86^, ^87^

Dermoscopic peculiarities are observed in some subtypes of actinic keratoses: Bowenoid actinic keratosis presents glomerular vessels regularly distributed along the lesion, differentiating it from Bowen's disease, whose vessels are irregularly distributed and grouped; hyperkeratotic actinic keratoses present a non-specific pattern due to hyperkeratosis, which prevents visualization of the underlying structures.^66^, ^88^

Dermoscopy of non-facial actinic keratoses includes erythema and superficial scales, sometimes accompanied by dotted vessels.^88^, ^89^ Furthermore, the erythematous pseudonetwork may eventually be found, although it is characteristic of facial lesions.^89^, ^90^

When the patient has multiple actinic keratoses, some authors describe that the lesions tend to follow the same dermoscopic pattern.^91^ This tendency occurs in relation to pigmentation: patients with higher phototypes tend to present multiple pigmented lesions, while those with lighter phototypes tend to have non-pigmented lesions.^91^

Dermoscopy may be useful for evaluating the progression of actinic keratoses to invasive SCC, as described by Zalaudek et al. (Table 3).^92^

**Table 3:** Progression model of actinic keratoses to invasive squamous cell carcinoma (SCC) based on dermoscopic analysis.

Clinical diagnosis	Dermatoscopic findings
Actinic keratosis	Red pseudonetwork
Actinic keratosis evolving to *in situ* SCC	Starburst pattern
*In situ* SCC	Yellowish opaque structures and dotted vessels
*In situ* SCC progressing to invasive SCC	White areas without structures, dotted and hairpin vessels
Minimally invasive SCC	Central keratin mass and vessels hairpin
Invasive SCC	Central keratin mass, ulceration, and irregular linear vessels

**Figure 4:**
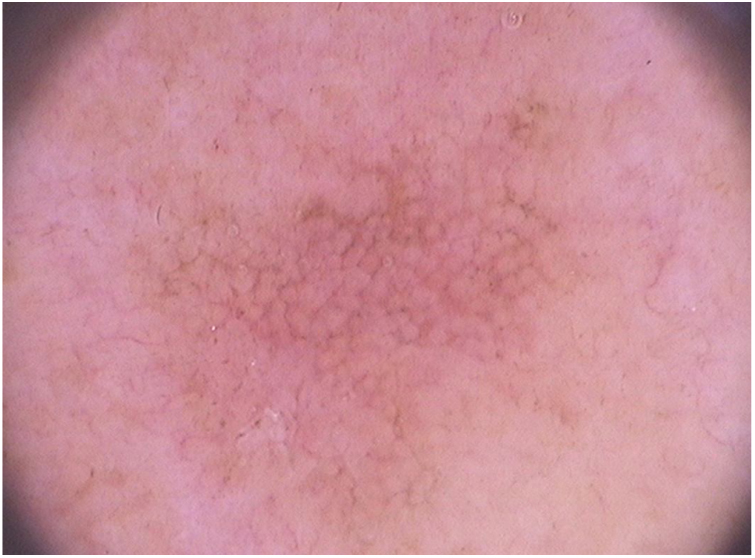
Dermoscopy of facial pigmented actinic keratosis (FotoFinder®, x20): a brownish pseudonetwork that spares follicular ostia is observed, in addition to surface scales and underlying vascular pseudonetwork.

In relation to dermoscopy of pigmented actinic keratoses, the main types of pigmentation are: brownish pseudonetwork, homogeneous, annular–granular, brownish or grayish pigmentation in spots and globules, and inner gray halo.^93^ In the face, pigmented pseudonetwork and granular–annular pigmentation predominate (Fig. 4).

**Figure 5:**
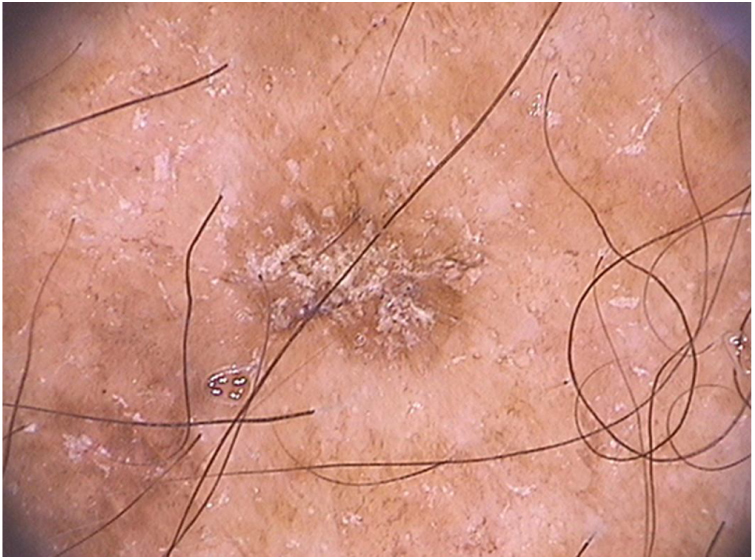
Dermoscopy of extra-facial pigmented actinic keratosis located on the forearm: homogenous brownish pigment with whitish scales on the lesion surface can be observed (FotoFinder®, x20).

Regarding non-facial pigmented actinic keratoses, they may present a reticular pattern with a delicate pigmented network, homogeneous pigmentation, or multiple irregular dots and globules of brownish to blue-gray color (Fig. 5).^66^

### Noninvasive imaging

#### Confocal microscopy

CM is another noninvasive, *in vivo* technique very helpful in examining equivocal lesions. The CM principle is based on the reflection, dispersion, and absorption of near-infrared light, allowing horizontal assessments of lesions at the level of the epidermis and superficial dermis.^94^, ^95^ Therefore, hyperkeratotic lesions do not present good visualization with CM due to the low penetrance of infrared light.^94^, ^96^ The main findings of actinic keratoses in CM include: superficial scales, which present as amorphous material with variable refractibility at the level of the stratum corneum, parakeratosis, presented as cells delimited by a whitish halo with a black center, and an irregular honeycomb pattern in the granular and spinous layer due to the presence of keratinocytes of varying sizes.^60^ Actinic keratoses and SCC by CM are differentiated by the dermal alterations present only in SCC, which include the presence of pleomorphic dermal cells.^94^

CM is also helpful in evaluating pigmented actinic keratoses.^93^ In addition to the structures mentioned before, it is also possible to observe increased epidermal thickness and intraepidermal dendritic cells, corresponding to Langerhans cells.^94^

#### Optical coherence tomography

Optical coherence tomography (OCT) is another *in vivo*, noninvasive imaging method with 86% sensitivity and 83% specificity for the diagnosis of actinic keratoses.^94^ The technique is based on the principle of interferometry, which uses infrared radiation and allows the visualization of epidermal layers, and adnexal and vascular structures.^94^ The images produced by OCT are biologically three-dimensional, with penetration of 500–1000 mm and lateral spreading of 4–6 mm.^97^ The main findings observed in actinic keratoses are thickening, and the strongest scattering comes from the stratum corneum due to parakeratosis, besides increasing the total thickness of epidermis, with well defined demarcation with the dermis.^97^ In hyperkeratotic actinic keratoses visualization with OCT is difficult.^97^

### Histopathological examination

The histopathological examination of actinic keratoses is characterized by atypical and pleomorphic keratinocytes in the basal layer of epidermis and by defective maturation of keratinocytes in superficial layers, with abnormal architecture of the epidermis. The number of mitoses is increased and polarity of keratinocytes is lost.^43^ The so-called “flag sign” is observed, due to the alternation of parakeratosis and hyperkeratosis in the stratum corneum, because the lesion spares the acrosyringium and the acrotrichium.^3^ Other possible findings include mild inflammatory infiltrate composed of lymphocytes and histiocytes, areas of acanthosis and acantholysis, and solar elastosis.^3^, ^67^, ^69^

### Immunohistochemical examination

Immunohistochemical examination is not routinely performed for the diagnosis of actinic keratoses, but may be useful in the differentiation of suspicious lesions, differentiating them from Bowen's or Paget's disease and melanoma.^98^ Anti-cytokeratin (CK) antibodies are the main antibodies used in the immunohistochemical panel of actinic keratoses, since keratins are the main proteins present in epidermis.^99^ Actinic keratoses tend to present CK5/8 positive histochemical panel in epidermis; moreover, some cases present positivity for CK15 and CK19, epidermal stem cell markers, and negativity for CK7.^98^, ^100^ Positivity for S100 favors the diagnosis of melanocytic lesions and can be helpful when differentiating actinic keratosis from melanoma.^101^

Furthermore, although they are not routinely used, markers of cell proliferation and cell cycle deregulation are observed in the epidermal layers.^102^, ^103^

## Differential diagnosis

The differential diagnoses of actinic keratoses include seborrheic keratosis, Bowen's disease, SCC, solar lentigo, stucco keratosis, basal cell carcinoma (BCC), porokeratosis, clear cell acanthoma, psoriasis, lupus erythematosus, lichen planus, and viral warts.^1^, ^90^ In pigmented facial actinic keratoses, the main differential diagnosis is lentigo maligna, although other benign lesions enter the differential diagnosis, such as solar lentigo, seborrheic keratosis, and lichenoid keratosis (Table 4).^93^, ^104^, ^105^, ^106^ The correct recognition of each of these differential diagnoses is essential, as they vary in relation to prognosis and necessary treatments.^106^ In this context, dermoscopy is fundamental, as it helps to differentiate them.

**Table 4:** Differential diagnoses of facial pigmented lesions by dermoscopy.

	Pigmented actinic keratosis	Lentigo maligna melanoma	Senile lentigo	Seborrheic keratosis	Lichenoid keratosis
Rhomboidal structures	+	+	−	−	+/−
Grayish-brown granulation	Uniform perifollicular distribution	Diffuse distribution	−	−	Diffuse distribution
Annular–granular pattern	+	+	−	−	+
Asymmetric follicular pigmentation	−	+	−	−	−
Homogeneous areas of pigment	−	+	−	−	−
Moth-eaten border	+/−	−	+	+	−
Jelly sign	−	−	+	+	−
Fingerprint-like structures	−	−	+	+	−

Adapted from: Ciudad C, et al. Diagnostic utility of dermoscopy in pigmented actinic keratosis (2011).^107^

Despite the difficult differential diagnosis between pigmented actinic keratoses and lentigo maligna on the face, dermoscopy may be useful. The presence of homogeneous areas (obliteration of follicles) is highly suggestive of lentigo maligna, whereas spared follicular structures and inner gray halo plus follicular plugs favor the hypothesis of pigmented actinic keratosis.^66^, ^108^

## Evolution and prognosis

Actinic keratoses may follow three different paths, the most relevant being the transformation into SCC. However, a significant portion of lesions remain stable throughout their evolution and may also involute spontaneously, although recurrences are frequent.^8^, ^109^, ^110^

The carcinogenic process in actinic keratoses follows the multi-stage carcinogenesis model: an initial mutation in a tumor suppressor gene leads to a precursor lesion, and later mutations in oncogenes lead to invasive properties.^111^ This progression can occur by the classical pathway, in which there is progression of keratinocytic dysplasia from more superficial epidermal layers toward the basal layer and the dermis,^3^ or by the differentiated pathway, a more aggressive and common pathway in which keratinocyte dysplasia located only in the basal epidermal layers evolves into invasive SCC by adnexal invasion.^112^

Data about the risk of malignant transformation of a single actinic keratosis ranges from 0.1% to 16%.^37^, ^109^, ^113^, ^114^, ^115^ In 10 years, the risk of malignant transformation of a single lesion is of about 10% for immunocompetent patients and 20% for immunocompromised patients.^67^, ^115^, ^116^ Thus, when assessing the risk of malignant transformation of actinic keratoses in patients with multiple lesions, the risk will be higher than that described for patients presenting a single lesions.^117^ Indeed, their risk of developing basal cell carcinoma (BCC) and SCC is higher than in the general population.^118^ In a population-based study in the United States (mean age of the analyzed population of 79 years), the risk of developing skin cancer in a patient with actinic keratoses was six-fold higher than the population without actinic keratoses (*p* < 0.001), both for NMSC and for melanoma.^119^ The study also showed that this risk was increased in the Caucasian population (*p* < 0.01), with the risk raising as patient age increased.^119^

According to some authors, 60–80% of SCCs develop in areas of actinic keratoses.^109^, ^114^, ^120^, ^121^ Some studies even suggest that time is a cofactor for the transformation of actinic keratoses into invasive lesions, with an average time to transformation for invasive lesions of 24.6 months (95% CI: 21.04–28.16 months).^39^, ^122^

A significant proportion of actinic keratoses remain stable throughout their course (63.1%); this is the most frequent evolution in the natural course of actinic keratoses.^37^ They often grow in size, quantity, or become more hyperkeratotic if not treated.^37^

Finally, a minority of lesions (from 20% to 23% in patients with a single lesion and from 0% to 7.2% in patients with field cancerization) may disappear spontaneously; however, lesional recurrence occurs in a significant portion of patients, affecting 57% of the cases.^110^ The proposed mechanism to explain spontaneous remission of actinic keratoses involves a sufficient immune response leading to lesion destruction; in addition, the reduction of UV radiation exposure could be involved.^37^ Moreover, spontaneous lesion regression rates are even lower in immunosuppressed patients.^123^

The evolution of actinic keratoses varies according to patient's clinical characteristics. Immunosuppressed individuals or patients with previous NMSC present a higher risk of malignant transformation of their actinic keratoses. In addition, in the same individual, actinic keratoses have dynamic characteristics: some lesions regress spontaneously while others evolve into invasive lesions; Identifying which lesions will develop into SCC is a challenge.^110^ Another important consideration is that patients diagnosed with actinic keratoses present a risk of developing new lesions in one year of 60%, demonstrating the chronicity of this pathology.^13^, ^37^

Patients with actinic keratoses usually have a good prognosis, as it is not directly associated with mortality; however, different degrees of morbidity associated with lesions treatment and with symptoms of the disease are frequent. In addition, the prognosis may vary in patients with more aggressive lesions and association with SCC. Moreover, the overall mortality for this neoplasm is around 3–4% of the cases.^124^ Furthermore, chronically photoexposed patients presenting actinic keratoses are at a higher risk of developing NMSC and melanoma; because of that, a higher level of surveillance for skin cancers is important in these individuals.^125^, ^126^, ^127^

## Treatment

Considering that actinic keratoses are potentially associated to malignant transformation and that it is not possible to predict which lesions it will occur, all lesions should be treated.^1^, ^9^, ^116^, ^128^

Some practices are essential in the management of patients with actinic keratoses: (1) regular total body skin examination, (2) assessment of the presence and treatment of field cancerization, (3) focused ablative methods for hyperkeratotic lesions or similar, (4) patient education regarding the chronic course of actinic keratoses, the need for photoprotection, and frequent treatments, and (5) regular skin self-examination by the patient.^129^

Therapeutic alternatives for actinic keratoses include several modalities. Basically, they can be divided into ablative or surgical methods and topical therapy (Table 5).^130^ The use of these methods in association or in sequence is common in the management of these patients.^131^ Treatment selection varies according to the clinical presentation, its location, and the number and extent of lesions; therefore, treatment should be individualized according to the needs of each patient.^132^ Notably, 25–75% of the patients treated will need retreatment within 12 months due to the appearance of new lesions, demonstrating the chronicity of this condition, even if field cancerization treatment has been performed.^127^ The worst recurrence rates are observed in patients submitted only to cryotherapy and the lowest rates are observed in those who have undergone field cancerization treatment.^133^

**Table 5:** Ablative-surgical and drug therapy modalities for actinic keratoses and their levels of evidence.

Modalities of surgical treatments	Topical and oral treatment modalities
Cryosurgery (recommendation grade A, level of evidence 1++)^131^	5-Fluorouracil (recommendation grade A, level of evidence 1++)^131^
	Imiquimod (recommendation grade A, level of evidence 1++)^131^
CO_2_ laser (recommendation grade B, level of evidence 1+)^131^	Ingenol mebutate (recommendation grade A, level of evidence 1+)^131^
	Photodynamic therapy (recommendation grade A, level of evidence 1+)^131^
Curettage and electrodessication (recommendation grade D, level of evidence 4)	Diclofenac (recommendation grade A, level of evidence 1+)^131^
	Topical retinoids (recommendation grade B, level of evidence 1+)^131^
Surgical exeresis (recommendation grade D, level of evidence 4)	Systemic therapies (recommendation grade C, level of evidence 2+)^131^

Degree of recommendation: A, at least one meta-analysis, systematic review or RCT 1+ and meta-analyses; B, group of 2++ studies with consistent results; C, group of 2+ studies with consistent results; D, evidence level 3 or 4, or formal consensus.

Level of evidence: 1++: high quality meta-analyses, systematic review of RCTs or RCTs with very low risk of bias; 1+: well-conducted meta-analyses, systematic review of RCTs or RCTs with low risk of bias; (1) meta-analysis, systematic review or RCT with high risk of bias; 2++: high quality systematic reviews, case–control or cohort studies. The case–control and cohort studies have a low risk of confounders, biases and a high probability of having a causal relationship. 2+: case–control studies or well-conducted cohorts with low risk of confounders and biases, and moderate likelihood of presenting causal relationship; (2) case–control studies or well-conducted cohorts with low confounders and biases, and high probability of not having a causal relationship; (3) non-analytical studies (case reports, case series); (4) expert opinion, formal consensus.

RCT, randomized clinical trial.

A large systematic review for the treatment of actinic keratoses concluded that 5-fluorouracil (5-FU), diclofenac, imiquimod, and ingenol mebutate (IM) may present similar efficacy.^134^ Gupta et al. evaluated the efficacy of topical therapies for actinic keratoses, and observed that the following options presented, respectively, decreasing rates: 5-FU 5%, 5-FU 0.5%, photodynamic therapy (photodynamic therapy (PDT)) with aminolevulinic acid (ALA), imiquimod, IM, methylaminolevulinic (MAL) PDT, cryotherapy, and diclofenac gel.^135^ Another meta-analysis observed that PDT with ALA was the most effective in achieving complete response; however, this meta-analysis might not reflect updated results, as it excluded more recent studies.^136^, ^137^

Classically, patients can be classified in four subgroups according to disease extension to define the best therapeutic modality to be used: patients with single lesions (<5 lesions per body area), with multiple lesions (six or more lesions per body area), those with areas of field cancerization, and immunosuppressed patients.^76^

### Topical treatments

#### 5-FU

5-FU is used for actinic keratosis in concentrations ranging from 0.5% to 5%. In Brazil, it is available commercially only at 5%. 5-FU acts by interfering in DNA synthesis through irreversible inactivation of thymidylate synthase; the final result is apoptosis of high proliferation cells, such as the actinic keratosis keratinocytes.^138^

5-FU 5% cream is recommended to be used twice daily over the lesional area for a period of two to four weeks. The treated area should not exceed 500 cm^2^ in a single treatment. Multiple areas should be managed sequentially. Side effects such as burning sensation, formation of crusts, erythema, vesiculation, erosion, pain, photosensitivity, pruritus, and ulceration are commonly expected; these effects are due the pharmacological effects of 5-FU and the patient should be aware of their occurrence. The perception of side effects beyond clinically detectable lesions demonstrates the potential of 5-FU in treating field cancerization areas and revealing subclinical actinic keratoses. The final treatment result is not evident until one to two months after treatment is complete.

A split-face study comparing 5-FU cream 0.5% once daily *vs.* 5-FU cream 5% twice daily applied during a four-week period observed similar efficacy in the percent of reduction of the number of actinic keratosis and in the percent of patients that achieved complete clearance of lesions; besides that, 5-FU at 0.5% concentration was superior in the reduction of total actinic keratoses and this concentration had a better tolerability profile.^139^ Complete response with 5-FU 5% is achieved in a range of 50–96% of the patients. It is sustained after one year, respectively, in 54% and 33% of patients with isolated lesions and for those with field cancerization.^133^, ^140^

The degree of recommendation for the use of 5-FU 0.5% for field cancerization of immunocompetent patients is strong; it is considered preferable to 5%. In immunocompromised patients, it is recommended to use 5-FU 5%.^76^ Nevertheless, in this scenario it has limited efficacy and the degree of recommendation for this specific population is also low.^76^

Preparations of 5-FU 0.5% combined with salicylic acid 10% are also described. In this case, salicylic acid acts as a keratolytic agent to increase the effect of 5-FU, and is indicated for the treatment of grade I and II actinic keratoses, used for a period of 6–12 weeks.^141^ The degree of recommendation of this combination is low for immunocompetent patients, both for isolated and for multiple lesions, including field cancerization.^76^

#### Imiquimod

Imiquimod is a synthetic compound from the imidazoquinoline family that acts as an immunomodulator. The medication acts as a tool-like receptor in the messenger RNA expression of immunomodulatory genes that induce cytokines production; as a result, innate and acquired immune response is stimulated, with increased antitumor and antiviral activities.^142^ Moreover, imiquimod activates pro-apoptotic pathways.^143^

Topical imiquimod for actinic keratoses may be used with distinct concentrations: 2.5%, 3.75%, and 5%. In Brazil, the product is available only in the latter form. Imiquimod 3.75% for actinic keratoses treatment is recommended to be used daily for two weeks, followed by a pause of two weeks, and then another cycle of two weeks.^144^ Imiquimod 5% for actinic keratoses treatment is recommended to be used two to three days a week, during a period of 4–16 weeks; after application, the product must remain on the skin for 8 h.^76^ The use of imiquimod 5% three times a week for four weeks was more effective than 5-FU 5% and cryotherapy.^132^ In one study, the complete remission rate after one year was 73% in the patients that used imiquimod.^133^

The degree of recommendation for treatment of field cancerization with imiquimod 3.75% is strong, while for imiquimod 5% the degree of recommendation is weak; this difference occurs due to the methodological quality of the studies.^76^

#### Ingenol mebutate

IM is derived from the *Euphorbia peplus* plant, and is available for commercial use in 0.015% concentrations for facial and scalp actinic keratoses treatment, indicated to be used for three consecutive days (one vial/day), and in a 0.05% concentration for use in non-facial areas for two consecutive days. IM has two mechanisms of action; both cytotoxic and immunomodulatory effects mediated by neutrophils occur.^145^ Expected adverse effects with treatment include erythema (94%), edema (79%), vesiculation (56%), formation of crusts (80%), desquamation (85%), erosion and ulceration (32%), with the highest intensity occurring four days after treatment completion in facial lesions and between the third and eighth day for non-facial lesions.^146^

Placebo-controlled studies showed a complete response rate of 37–44% in facial and scalp actinic keratoses with IM and of 39–42% in trunk and limbs lesions. This response was maintained for 12 months in 46.1% of the facial lesions and 44% of the non-facial lesions treated.^146^ In addition, only three of the 171 patients evaluated in these studies had mild non-treatment-related side effects.^147^ In a longitudinal study in the Brazilian population with 27 patients presenting actinic keratoses treated with IM, a complete response was observed in 53.8% of the actinic facial keratoses treated and in 42.8% of the non-facial lesions; in addition, the treatment was well tolerated.^148^

Besides placebo controlled studies there are a few reports comparing the use of IM with other therapeutic modalities, such as diclofenac sodium gel and 5-FU. A phase IV study comparing IM with diclofenac gel for treatment of grade I and II actinic keratoses on the face and scalp observed complete response after a cycle of treatment with IM of 34% of treated lesions, and of 23% after 90 days of use of diclofenac gel; after a second treatment, the complete response rates were 53% for the IM group and 45% for the diclofenac group (*p* < 0.001).^149^ Another study compared the use of IM for three consecutive days in facial actinic keratoses with 5-FU, applied twice daily for four weeks, in relation to adverse effects profile and safety profile. Both treatments were safe and the withdrawal rate was similar between the treated groups; however, the peak and the duration of the local cutaneous reactions differed between the groups: the peak of IM cutaneous reactions occurred in four days, with an average duration of 15 days, and with 5-FU the peak occurred in 29 days and symptoms lasted until day 36.^150^

The degree of recommendation for IM is strong for the treatment of field cancerization in immunocompetent patients, and it is less valuable for patients with localized lesions.^76^ Due to the lack of studies, it is not possible to provide a recommendation for IM use in immunosuppressed patients; however, Mühlstädt et al. described a single case of an immunosuppressed renal transplant patient with partial response to IM for facial actinic keratoses.^151^

#### PDT

PDT consists of using a photosensitizing agent and a light source at a specific wavelength to produce reactive oxygen species, which then destroy target lesions through a photochemical reaction.^152^, ^153^ This reaction is achieved by the application of 5-aminolevulinic acid (5-ALA) or methylaminolevulinate (MAL), which are precursors of photoactive metabolites (protoporphyrin IX). These metabolites accumulate in neoplastic cells and, when activated by visible light, lead to the formation of reactive oxygen species and oxygen singlets.^152^ As a result, these reactive oxygen species initiate a biochemical cascade of events that induce death of the target cell by apoptosis or necrosis and an immunomodulatory effect.^154^

The photochemical reaction for each photosensitizing agent occurs after irradiation with a light source at a specific wavelength in the visible light spectrum.^155^ There are four peaks of porphyrin absorption in this spectrum; the largest is in the blue light spectrum, at 410 nm, with smaller peaks at 540 nm, 580 nm, and 635 nm.^156^ The red light (625–740 nm) penetrates deeper than the blue, reaching up to 3 mm, therefore it is preferable in treating thicker lesions. The blue light spectrum (440–485 nm) reaches a depth of 1–2 mm and is generally used to treat superficial lesions.^157^ Light emitting diode (LED) devices are the most used light sources for PDT, and they are considered the gold standard, but intense pulsed light, halogen or xenon light, argon laser, Nd:YAG laser, and pulsed dye laser can also be used.^158^ Before the application of the photosensitive agent on the area to be treated, superficial curettage of the lesions is recommended.^156^ In addition, some methods can be used to increase penetration of the photosensitizing agent, such as microneedling, ablative fractional laser, and calcipotriol application.^156^, ^159^, ^160^ According to a Cochrane Review for the management of actinic keratoses, PDT with ALA or MAL is effective whether either using red LED light or blue LED light, with similar efficacy.^133^

Currently, MAL is the only photosensitizing agent available commercially for PDT in Brazil. In the conventional PDT protocol, a thin layer of 1 mm of the product should be applied to the area to be treated, which is occluded for 3 h, then the area should be cleansed and irradiated with the chosen light source. The main randomized controlled trials evaluating the full response rate three months after performing ALA PDT demonstrated that 69–91% of the patients treated achieved complete clearance of the lesions.^161^ Complete remission with MAL PDT at three months occurs in 90% of the cases.^162^ In addition, excellent cosmetic results are observed in 91–98% of the patients treated.^163^ One year after PDT, one-quarter of the patients presented lesion recurrences (24%).^164^ PDT possesses a strong recommendation level for the treatment of actinic keratoses and field cancerization.^165^, ^166^ However, side effects are frequent. About 20% of patients complain of severe pain (grade of pain over 6 on a scale of 0–10) during LED emission, and remain with intense erythema and desquamation for up to 21 days.^167^, ^168^, ^169^ A limitation of PDT is its use in pigmented lesions, which diminish the effectiveness of the photochemical reaction, since the melanic pigment competes with protoporphyrin IX in light absorption, reducing the desired photodynamic effect.^170^

More recently, daylight PDT has been described as a technique with similar response rates as conventional PDT, but fewer irradiation-related side effects.^171^ The technique consists of the application of MAL cream and after 30 min of incubation, without occlusion, the patient is exposed to daylight for approximately 2 h to allow the activation of MAL by visible light, ranging from 380 to 740 nm.^172^ Daylight PDT is mainly recommended for the treatment of non-pigmented grade I and II actinic keratoses.^172^ A series of 20 Brazilian patients with actinic keratoses of the face and scalp submitted to the technique showed excellent tolerability for patients; 80% reported minimal discomfort during the irradiation period.^171^ Studies comparing conventional PDT with daylight PDT demonstrated similar effectiveness and safety of both techniques for treatment of grade I and II actinic keratoses of the face and scalp.^173^, ^174^, ^175^ For treatment of multiple lesions and field cancerization, both ALA PDT and MAL PDT have a strong degree of recommendation. Again, due to the lack of clinical trials in immunocompromised patients and to the challenge of treating actinic keratoses in these patients, PDT has a weak recommendation degree for this indication.^76^

#### Diclofenac

The use of 3% diclofenac gel, a non-steroidal anti-inflammatory, plus 2.5% hyaluronic acid, used to optimize the permeation of diclofenac in the epidermis, for the treatment of actinic keratoses is recommended to be applied twice daily for a minimum period of 60–90 days.^176^ The mechanism of action proposed for this therapy is the inhibition of cyclooxygenase-2 (COX-2), which leads to a reduction in prostaglandin synthesis and inhibition of cell differentiation and angiogenesis, induction of apoptosis, and changes in cell proliferation.^177^ Diclofenac also activates nuclear hormone receptors involved in cell differentiation and apoptosis.^177^ The use of diclofenac gel for 90 days results in complete lesions clearance in 50% of patients treated and, if used for 60 days, in 33% of patients.^176^ Regarding long-term efficacy, a recent study observed sustained remission one year after treatment in 95% of patients who initially presented complete response, and in 45% of the immunosuppressed patients treated with 90 days of diclofenac gel.^178^ Furthermore, Segatto et al. compared the use of 5-FU 5% twice daily for four weeks *vs.* diclofenac gel 3% twice daily for 12 weeks; although the reduction in the total number of actinic keratoses was significantly higher in the 5-FU group (*p* < 0.001), there was a greater tolerance and a lower number of adverse effects in the diclofenac group (93.3% for 5-FU *vs.* 38.4% for diclofenac, *p* = 0.008).^179^ One possible limitation for actinic keratoses treatment with diclofenac gel is patient adherence to treatment, as its duration is between 60 and 90 days (*p* = 0.008).^76^

Therefore, diclofenac gel treatment may be an option for patients who have not tolerated other topical treatment modalities for actinic keratoses.^180^, ^181^

### Ablative-surgical treatments

#### Curettage

The use of curettage under local anesthesia for treatment of actinic keratoses can be performed in isolation or in association with electrodessication, which appears to increase the resolution of potential remaining dysplastic cells and also to achieve hemostasis. An alternative to electrodessication is cryotherapy.^130^ As a monotherapy, curettage is especially indicated for patients with few lesions, especially hyperkeratotic actinic keratoses. The method is frequently used in the setting of patients with large clinical variability of keratoses as a complementary therapy for lesions resistant to field cancerization therapy, in addition to allowing the collection of material for histopathological analysis.^130^ Disadvantages regarding the method include the need for local anesthesia, the healing time, which can be prolonged when lesions are treated especially in the lower limbs, and the risk of dyspigmentation symptoms in the treated area.^76^, ^130^ Although curettage is widely performed in daily practice, the lack of randomized clinical trials evaluating the subject results in a low degree of recommendation of the procedure for actinic keratoses treatment.^76^

#### Cryotherapy

Cryotherapy is a destructive method used for the isolated treatment of actinic keratoses, which uses liquid nitrogen (LN) to achieve tissue freezing and thawing processes, leading to tissue destruction.^182^ Cryotherapy is the treatment of choice in patients presenting isolated or small numbers of lesions without field cancerization. The technique consists in applying LN as a spray or in an object that exerts direct pressure on the skin, such as a swab.^182^ LN temperature is −196 °C, and ideally it reaches approximately −50 °C in contact with the skin. The freezing area can reach up to 10 mm in depth, according to the duration and distance from the skin on which it is applied.^183^

The efficacy of the technique can range from 69% of the lesions achieving complete clearance with freezing time greater than 5 s to 83% with more than 20 s of freezing.^184^ Histological changes after a single cycle of cryotherapy with a 10-s duration include reduction in keratinocyte atypia, in epidermal and stratum corneum thickness, and in lymphocytic infiltrate.^185^ The available studies considering single-cycle or double-cycle freezing cryotherapy compared the effectiveness with photodynamic therapy (PDT); one study performed single-cycle (10’) sessions of cryotherapy, repeated every three months until complete clearance was achieved by each patient, and observed a complete response in 85% of the cases treated after 12 months.^166^ Another study used a double cryotherapy cycle (freezing time not reported) in a single session and achieved complete response in 88% of treated cases at 24 months of follow-up.^186^ When comparing MAL-photodynamic therapy (PDT) *vs.* cryotherapy (double cycling) in actinic keratoses, at 12 months of follow-up the complete response with photodynamic therapy (PDT) was 89.1% *vs.* 86.1% with cryotherapy, with no statistical difference between the groups.^187^

Adverse effects described involve pain and burning sensation during the application, as well as erythema, edema, vesiculation during the following days, and residual hypopigmentation.^184^ Due to the latter condition, it is important to be cautious with the freezing time in patients with high skin phototypes. Despite its widespread use in dermatological practice, sustained complete remission of cryotherapy in patients with isolated lesions after one year of follow-up is lower (only 28%) than that seen with 5-FU (54%) and imiquimod (73%). This is precisely because some patients have pre-clinical symptoms in the vicinity of the lesions treated.^133^

It is a therapeutic modality with low cost, easy accessibility, and good adherence by the patient. In addition, it can be used in localized lesions in association with field cancerization treatments.^188^, ^189^ Disadvantages include the fact that the method does not allow treatment of field cancerization, the discomfort at the time of application, and the recovery time. The degree of recommendation for the treatment of localized lesions with cryotherapy in immunocompetent patients is strong, whereas in immunosuppressed patients the effect is limited.^76^

#### CO_2_ laser

Lasers induce coagulative necrosis, ablation, and hyperthermia, which lead to lesional destruction. A single session of non-fractional CO_2_ laser could be used to remove superficial lesions on the epidermis, such as actinic keratoses. The 10,600 nm CO_2_ non-fractional laser has a wavelength absorbed by water, resulting in non-specific tissue destruction. Therefore, non-fractional CO_2_ laser can be used for field cancerization treatment or for localized lesion destruction. For localized lesions, complete lesion clearance results in the first months are similar to those obtained with cryotherapy (72.8% in the laser group *vs.* 78% for cryotherapy); however, in long-term follow-up, lesions treated with CO_2_ laser present lower sustained response rates: only 37% of the patients treated with laser remain without lesions *vs.* 66.8% of those treated with cryotherapy.^190^ Furthermore, because the technique is operator-dependent, different levels of expertise with the technique may influence the results.^76^ In addition, there is a risk of secondary infection, esthetical scars, and dyschromia. Because of the increased risk of infection in immunosuppressed patients, CO_2_ laser is not recommended for the treatment of field cancerization and should be used only for localized lesions in these patients.^76^ Although the use of CO_2_ laser can be considered as an option for actinic keratoses treatment, the degree of recommendation for its use in immunocompetent patients is weak.^191^

## Prevention

### Topical retinoids

One of the first studies to report the benefits of topical retinoids for patients with actinic keratoses dates back to 1970, a case series of 60 patients that reported benefits of tretinoin use at 0.1–0.3%, for a reduction in actinic keratoses scores of about 50%.^192^ Subsequent studies have shown that tretinoin at a lower concentration (0.05%) was not as effective, with a maximum reduction in the number of actinic keratoses of 45%.^193^, ^194^ However, despite these initial positive results, more recent studies evaluating the use of topical retinoids in a larger sample (>1000 individuals) have not been able to demonstrate their efficacy in reducing the occurrence of SCC and basal cell carcinoma (BCC) in patients at risk; besides, no benefit in reducing the number of actinic keratoses was observed.^195^, ^196^

### Serial peelings

Some studies have described the effect of serial peelings with glycolic acid, trichloroacetic acid (TCA), and salicylic acid in animal models previously exposed to UV radiation; they observed reduction of mutated p53 and expression of COX-2 mRNA, demonstrating a possible role in tumor prevention.^197^, ^198^, ^199^ In humans, there are few studies with high level of evidence on the subject. A split-face study including 15 patients with facial actinic keratoses a single session of Jessner's peel plus TCA 35% achieved similar effectiveness to the use of 5-FU twice daily for three weeks.^200^ There was a reduction of 75% in the total number of lesions in both groups, in addition to a similar reduction between treatments in keratinocytic atypia, parakeratosis, hyperkeratosis, and inflammation in histopathological analysis.

More recently, the association of glycolic acid or Jessner's peel with 5-FU 5% at fortnightly intervals demonstrated effectiveness for treatment of field cancerization^201^; 31 patients were submitted to the sessions until complete lesion remission or until completing ten sessions. The treatment was effective and showed good tolerability; moreover, only five patients presented relapses after 36 months of follow-up.

### Oral retinoids

Oral retinoids, synthetic derivatives of vitamin A, are used for chemoprevention of NMSC in high-risk patients for both immunocompetent and immunosuppressed patients, including patients with genodermatoses, such as xeroderma pigmentosum.^202^, ^203^, ^204^ The main medications described in the studies are acitretin, etretinate, and isotretinoin; among them, acitretin has the greatest degree of evidence regarding its protective effect.^205^ Several mechanisms of action are proposed to explain the chemopreventive effect of retinoids, including immunomodulation, apoptosis, promotion of cell differentiation, and inhibition of keratinization and cell proliferation.^202^ The first randomized clinical trial evaluating the use of 5 mg etretinate three times a week in 100 patients with actinic keratoses for a period of two months observed complete or partial remission of actinic keratoses in 84% of the treated patients (37 of 44 patients) *vs.* 5% (2 of 42 patients) in the placebo group.^206^

Another clinical trial with acitretin 30 mg daily for six months in 44 transplant recipients patients observed a 13.4% reduction in actinic keratoses in the treated group *vs.* an increase of 28.2% in the number of actinic keratoses in the placebo group.^207^ In addition, there was a reduction in the appearance of new SCCs in the acitretin group: only two of the 19 patients in the intervention group (11%) developed SCCs *vs.* nine of the 19 patients of the placebo group (47%) (the relative risk reduction of developing SCCs was of 78% for patients taking acitretin).^205^, ^207^ Smit et al. evaluated the use of acitretin 0.4 mg/kg/day for three months in 33 renal transplant recipients and performed histological and immunohistochemical analysis; modifications observed in actinic keratoses were reduction of epidermal thickness (*p* < 0.002) and normalization of the K10 keratinization pattern (*p* < 0.02). However, there was no change in cell proliferation, which could explain the early recurrence of actinic keratoses after acitretin discontinuation.^208^ Data on the optimal dose and duration of treatment are not defined in the literature.^203^

### Oral nicotinamide

Nicotinamide, the amide form of vitamin B3, is a cofactor for ATP production that prevents ATP depletion and glycolytic blockade induced by UV radiation, and thus assists in DNA repair. In addition, nicotinamide reduces UV-induced immunosuppression without altering basal immunity. Studies for the prevention of actinic keratoses are sparse, and its use for this purpose is still debated.

The use of nicotinamide as a chemoprotective agent to reduce the appearance of new lesions of NMSC and actinic keratoses in high-risk patients, a 1 g daily dose of the medication divided into two doses, was considered effective. After 12 months of follow-up, the rates of onset of new lesions in the patients receiving nicotinamide were 23% lower compared to the placebo group (*p* = 0.02), with reduction of both new basal cell carcinomas (20% reduction) and SCCs (30% reduction), as well as actinic keratoses (13%). The protective effect was maintained only during the use of the medication, which presented a good safety profile. However, subsequent studies are needed to confirm the reproducibility of the beneficial effects found, as well as the appropriate treatment duration.^209^

### Photoprotection

All patients with actinic keratosis should be advised regarding physical photoprotection and the use of sunscreens as an adjuvant to the treatment, and to prevent the onset of new lesions, regardless the type of treatment proposed.^132^, ^210^ Regular use of sunscreen with a sun protection factor (SPF) over 15 reduces the development of new actinic keratoses in immunocompetent patients, ranging from a 50% reduction in the number of new lesions in one year (study using SPF 29) and 37% in two years (study using SPF 16) (*p* < 0.05).^211^, ^212^ In addition, patients who benefit the most from sunscreen use to slow the development of new actinic keratoses include young patients who have not had previous NMSC and those who tan after sunbathing (phototypes ≥III).^211^ For immunocompromised patients, a case–control study with 120 immunosuppressed patients followed for 24 months observed that the use of broad spectrum SPF 50 sunscreen significantly reduced the appearance of new lesions in the intervention group (*p* < 0.05).^56^ Moreover, the effect of daily sunscreen use on spontaneous regression of actinic keratoses is also observed in both immunocompetent and immunosuppressed patients; this regression is higher than that observed in the lesions of patients who do not use sunscreen.^56^, ^210^, ^212^, ^213^ In addition, daily use of sunscreen reduces the incidence of new SCCs (*p* < 0.01).^56^, ^213^

More recently, studies using photolyases, broad-spectrum photoprotective cellular DNA repair enzymes that activated when the patient is exposed to visible blue light, appear to demonstrate benefit for their use in patients with actinic keratoses and field cancerization.^214^, ^215^ In a study with the use of photolyase in patients submitted to PDT, nine months after the procedure, none of the 15 patients in the photolyase group required a new field cancerization treatment *vs.* 67% (*n* = 10) of the 15 patients in the group that used regular sunscreen with SPF 50.^215^

## Final considerations

Actinic keratoses represent a cutaneous condition with an impact on quality of life, and they are an important precursor of cutaneous neoplasias.^92^ For this reason, the recognition and prompt treatment of such lesions are of extreme importance, since progression of the disease can be prevented. Thus, clinical and non-invasive imaging techniques, especially dermoscopy, can help with this task. Therapeutic options available are extensive and the choice of the best alternative should be individualized within the context of each patient and, whenever possible, based on scientific evidence.

## Financial support

None declared.

## Authors’ contribution

Clarissa Prieto Herman Reinehr: Approval of the final version of the manuscript; conception and planning of the study; composition of the manuscript; critical review of the literature.

Renato Marchiori Bakos: Approval of the final version of the manuscript; conception and planning of the study; participation in the study design; critical review of the manuscript.

## Conflicts of interest

None declared.

## Figures and Tables

**Table:** 


*1. Select the incorrect statement:*
a) In Brazil, actinic keratoses represent the fourth most frequent dermatological diagnosis.
b) The highest prevalence of actinic keratoses in Brazil occurs in the Southeast region.
c) In the international scenario, actinic keratoses have a higher prevalence in Australia than in Europe.
d) In patients over 65 years of age, actinic keratoses represent the main reason for dermatological consultation in Brazil.
*2. Regarding the effect of UVA ultraviolet radiation on carcinogenesis, it is correct to state that:*
a) It is responsible for changes in the telomerase gene.
b) It promotes mutations in the p53 protein.
c) It promotes DNA mutations with guanine (G) replacement by thymine (T).
d) It promotes errors in the repair of cyclobutane pyrimidine dimers and in the production of 6–4 photoproducts.
*3. Additional features considered to be at risk for the development of actinic keratoses include:*
a) Telangiectasias in the face and ephelides.
b) Solar elastosis and cutis rhomboidalis nuchae.
c) Alternatives a and b are incorrect.
d) Alternatives a and b are correct.
*4. Regarding the preferential location of actinic keratoses, indicate the correct alternative:*
a) 75% of the lesions are located in the lower limbs and in the face.
b) They are localized in photoexposed areas, such as face, scalp in bald individuals, cervix, cervical region, shoulders, forearms, and back of the hands.
c) Alternatives a and b are correct.
d) No alternative is correct.
*5. The following is included among the histopathological findings of actinic keratosis:*
a) Flag sign: alternating parakeratosis and orthokeratosis in the stratum corneum.
b) Parakeratosis occurs over the acrosyringium and acrotrichium.
c) There is no loss of polarity of keratinocytes.
d) Since it is a pre-neoplastic lesion, there is no atypia of keratinocytes.
*6. In relation to the concept of field cancerization, it is correct to state that:*
a) The concept was supplanted by the multi-stage carcinogenesis model.
b) It occurs only in patients with genodermatoses, such as xeroderma pigmentosum and Bloom's syndrome.
c) It is characterized by pre-neoplastic alterations of the epithelium after long exposure to UV radiation and consists of lesions in different phases.
d) It concerns multiple actinic keratoses, well delimited and easy-to-count, in a certain body area.
*7. Regarding dermoscopy of facial actinic keratoses, it is correct to state that:*
a) It is a sensitive but not very specific method for the diagnosis of actinic keratoses.
b) It is a high sensitivity and high specificity method for the diagnosis of actinic keratoses.
c) The method is not used due to the complexity of execution.
d) It is not very sensitive, although highly specific for the diagnosis of actinic keratoses.
*8. About the evolution of actinic keratoses for SCC, it is incorrect to state that:*
a) 60–80% of SCCs develop in areas of actinic keratoses.
b) The risk of progression to SCC is similar in immunocompetent and immunocompromised patients.
c) The mean time of transformation of actinic keratoses to invasive lesions is 24.6 months.
d) The multi-stage carcinogenesis model is applied to explain the malignant transformation of actinic keratoses.
*9. Regarding the use of 5-FU in the treatment of actinic keratoses, the following is incorrect:*
a) Regarding tolerability, the 5% concentration was preferred by the patients in the studies.
b) 5-FU acts through interference in DNA synthesis by irreversible inactivation of thymidylate synthetase.
c) The degree of recommendation for use of 5-FU at 0.5% for cancer treatment field treatment in immunocompetent patients is strong.
d) The use of 5-FU at 0.5 associated with 10% salicylic acid increases the permeation of 5-FU.
*10. In relation to the preventive methods for actinic keratoses and non-melanoma skin cancer, it is correct to state that:*
a) Topical retinoids are effective and safe for reducing the number of new SCCs and basal cell carcinoma (BCC)s, and reduce the number of actinic keratoses after three months of use.
b) Among systemic retinoids, isotretinoin has been shown to be the most effective in reducing the number of new SCCs and basal cell carcinoma in high-risk patients.
c) Oral nicotinamide has been described as promising agent for the development of non-melanoma skin cancer and actinic keratosis in high-risk patients.
d) The use of sunscreen has benefits only in the prevention of new actinic keratoses.

**Table:** 

1. c	3. a	5. d	7. a	9. a
2. b	4. c	6. c	8. b	10. d

## CME Questions

Answers:

Albinism: epidemiology, genetics, cutaneous characterization, psychosocial factors. An Bras Dermatol. 2019;94(5):503–520.
